# Hypercalcemic Crisis Induced by Hyperthyroidism of Graves' Disease: A Case Report

**DOI:** 10.1002/ccr3.73882

**Published:** 2026-11-05

**Authors:** Suxin Wang, Jing Wang

**Affiliations:** ^1^ Department of Endocrinology Sheyang County Peoples' Hospital Sheyang Jiangsu China

**Keywords:** graves' disease, hypercalcemia, hypercalcemic crisis, hyperthyroidism

## Abstract

Hyperthyroidism of Graves' disease (GD) is a rare but important cause of hypercalcemic crisis. In patients with hypercalcemia of unknown etiology, hyperthyroidism of Graves' disease should be considered.

**Abbreviations**
AFP: alpha fetoprotein
ALP: serum alkaline phosphatase
BMI: body mass index
CA125: carbohydrate antigen 125
CA15‐3: carbohydrate antigen 15‐3
CA19‐9: carbohydrate antigen 19‐9
CEA: carcinoembryonic antigen
C‐TIRADS: Chinese Thyroid Imaging Reporting and Data System
CYFRA 21‐1: cytokeratin 19 fragment
ECG: electrocardiogram
FT3: free T3
FT4: free T4
GD: Graves' disease
IgA: Immunoglobulin A
IgG: Immunoglobulin G
IgM: Immunoglobulin M
IL‐6: interleukin‐6
NSE: neuron‐specific enolase
PTH: parathyroid hormone
SCCA: squamous cell carcinoma antigen
SPE: Serum protein electrophoresis
TRAb: TSH receptor antibody
TSH: thyroid stimulation hormone
Wnt: wingless/integrated

## 1 Introduction

By the excess thyroid hormone, hyperthyroidism leads to a myriad of symptoms, such as heat intolerance, sweating, palpitation, tremor and weight loss. It impacts roughly 0.2%–1.4% of people around the world [1, 2]. The most common etiology of hyperthyroidism is Graves' disease (GD) [3]. Hyperthyroidism is considered to promote bone resorption more than bone formation, which results in osteoporosis [4]. Bone resorption can increase the serum calcium. The serum calcium seldom exceeds 3.0 mmol/L in hyperthyroidism induced hypercalcemia. Hypercalcemic crisis, in which the serum calcium level usually exceeds 3.5 mmol/L, is quite rare [5, 6, 7]. We report a case of hypercalcemic crisis induced by hyperthyroidism.

## 2 Case Presentation

A 57‐year‐old female presented to our hospital with anorexia, nausea, and vomiting. At the outpatient of gastroenterology, she accepted the gastroscopy; the result was erosive gastritis. Her electrocardiogram (ECG) was sinus tachycardia (117/min). She was taken rabeprazole after consultation with the physician, but her symptoms did not improve. Then she came to our hospital for further consultation. Her serum electrolytes were examined. The serum calcium was as high as 3.62 mmol/L (normal range: 2.10–2.60 mmol/L), with normal sodium, potassium, and phosphorus. The serum albumin was also normal. The serum calcium was a critical value; she was referred to the emergency department for immediate care. She was treated with normal saline (0.9% NaCl) and furosemide. After the treatment, her serum calcium decreased to 3.40 mmol/L. Then she was admitted for observation in the emergency resuscitation room. The next day she was admitted to our department. Her body temperature was 36.6°C, heart rate 109/min, blood pressure 130/80 mmHg. No obvious exophthalmos, no widening of the palpebral fissure, no goiter, no tenderness in the thyroid region, but tremor could be checked in both hands. Her body mass index (BMI) was 21.50 kg/m^2^. She had a weight loss of 10 kg in the month before admission. She additionally complained of constipation, thirst, polyuria, and irritability, but denied abdominal pain, back pain, or heat intolerance.

## 3 Investigations

To explore the etiology of hypercalcemia, we conducted further examinations. Blood investigations are presented in Table 1. Decreased parathyroid hormone (PTH) means the parathyroid function was inhibited by the hypercalcemia. Primary hyperparathyroidism was ruled out. According to the elevated free T3 (FT3) and free T4 (FT4), reduced thyroid stimulation hormone (TSH) and positive TSH receptor antibody (TRAb), GD was confirmed. Ultrasonography of the thyroid showed uniform echogenicity with a cystic nodule on the left lobe (Chinese Thyroid Imaging Reporting and Data System, C‐TIRADS II) (Figure 1). To investigate whether hypercalcemia was caused by tumor‐related diseases, ultrasound of the breasts, plain scan CT of the neck, thorax, abdomen and pelvis were done. The results were normal. No signs of bone destruction were mentioned in the CT examinations. Tumor markers were also tested, and the results were normal. These ruled out malignancy. The cortisol was normal, so adrenal insufficiency was excluded. We also detected immunoglobulin, such as IgA, IgG, IgM and serum protein electrophoresis (SPE) including β1‐globulin, β2‐globulin, α1‐globulin, α2‐globulin, γ‐globulin, M protein. Peripheral blood smear was done. These excluded multiple myeloma. In addition, dual‐energy X‐ray absorptiometry was done. Detection of lumbar spine (T score, L1: −1.7; L2: −2.7; L3: −2.7; L4: −2.0; total lumbar spine: −2.3), femoral neck (T score: −1.4), and whole femoral (T score: −1.1) revealed osteopenia.

**TABLE 1:** Blood investigations of the patient.

Test	Normal range	The first time	At admission	3rd day	10th day	1 week (follow‐up)	1 month	2 month
Serum calcium	2.10–2.60 mmol/L	3.62	3.19	3.33	2.13	1.91	2.07	2.29
Serum phosphorus	0.80–1.45 mmol/L	1.17	1.05					
Serum PTH	12.0–88.0 pg/mL		3.85			15.3		51.27
25‐OH‐D	≥ 50 nmol/L		51.74					
ALP	35–135U/L		148.9					
Serum FT3	2.0–4.4 pg/mL		13.9		4.3		2.59	4.59
Serum FT4	9.3–17.0 ng/L		44.7		25.3		6.49	8.23
Serum TSH	0.27–4.20 uIU/mL		< 0.005		< 0.005		< 0.005	0.0277
TRAb	0–1.75 IU/L		30.2				34.6	29.9
TgAb	< 115 IU/mL		13.9					
TPOAb	< 34 IU/mL		13.7					
Blood glucose	3.89–6.11 mmol/L		5.67					
Serum uric acid	155–357 μmol/L		684.5			392.3		
Serum creatinine	35–97 μmol/L		69.1					
Twenty‐four hour urine calcium	2.5–7.5 mmol/24h		3.54					
Cortisol (8 am)	4.82–19.5 μg/dL		8.09					
CEA	0–5 ug/L		0.492					
AFP	0–7 ng/mL		3.72					
CA19‐9	0–34U/mL		3.84					
CA125	0.7–4.0 g/L		7.31					
CA15‐3	7.0–16.0 g/L		8.02					
SCC	0–3.0 ng/mL		0.706					
CYFRA 21‐1	0–3.3 ug/L		2.9					
NSE	0–16.3 ug/L		12.4					
IgA	0.7–4.0 g/L		3.45					
IgG	7.0–16.0 g/L		15.8					
IgM	0.4–2.3 g/L		1.25					
SPE								
β1‐globulin	4.7%–7.2%		5.60%					
β2‐globulin	3.2%–6.5%		8.40%					
albumin	55.8%–66.1%		51.30%					
α1‐globulin	2.9%–4.9%		4.40%					
α2‐globulin	7.1%–11.8%		9.20%					
γ‐globulin	11.1%–18.8%		21.10%					
M protein	Negative		Negative					

Abbreviations: 25‐OH‐D,25‐hydroxy vitamin D; AFP, alpha fetoprotein; ALP, serum alkaline phosphatase; CA125, carbohydrate antigen 125; CA15‐3, carbohydrate antigen 15–3; CA19‐9, carbohydrate antigen 19–9; CEA, carcinoembryonic antigen; CYFRA 21‐1, cytokeratin 19 fragment; IgA, immunoglobulin A; IgG, immunoglobulin G; IgM, immunoglobulin M; NSE, neuron‐specific enolase; SCCA, squamous cell carcinoma antigen; SPE, serum protein electrophoresis; TgAb, antithyroid globulin antibody; TPOAb, antithyroid peroxidase antibody; TRAb, TSH receptor antibody.

**FIGURE 1:**
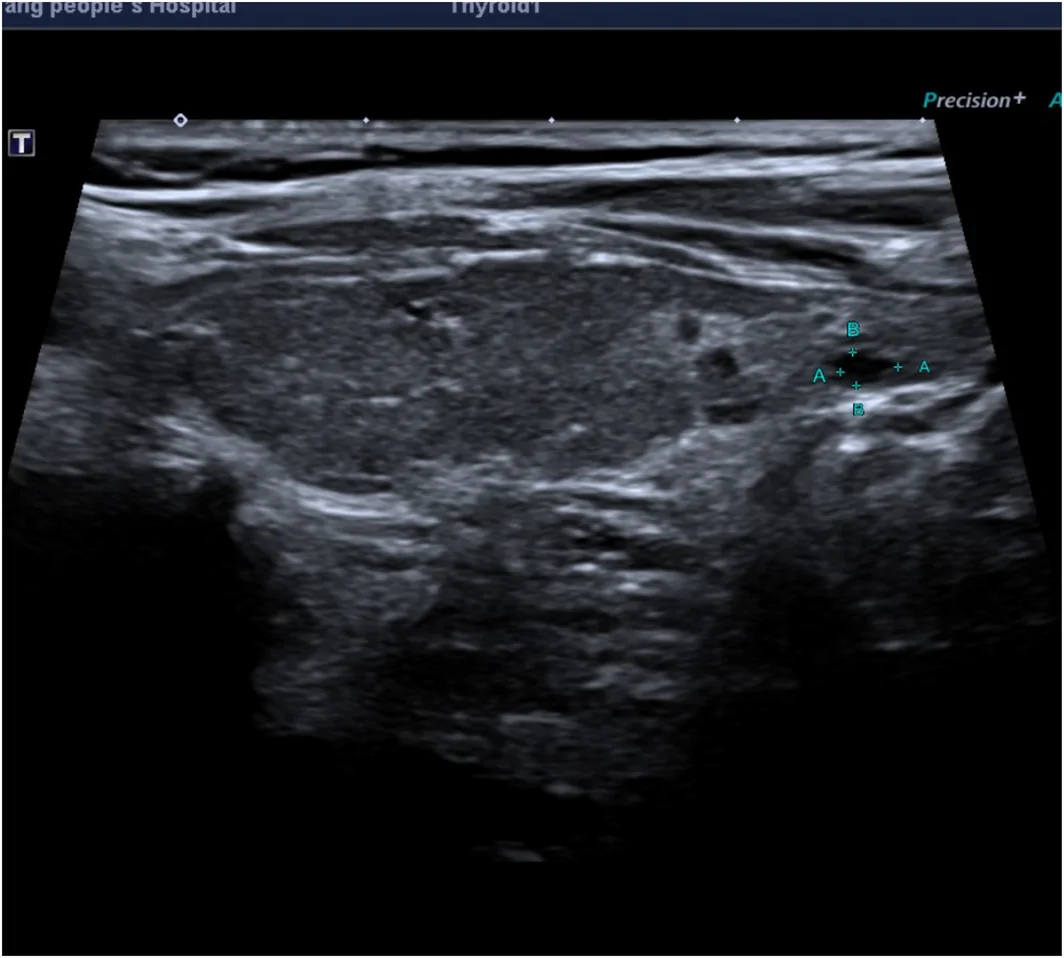
Thyroid ultrasound of the patient. Ultrasonography of the thyroid showed a cystic nodule on the left lobe.

## 4 Differential Diagnosis

Based on investigation results, we ruled out other PTH‐independent causes of hypercalcemia. We diagnosed her hypercalcemia as being induced by the hyperthyroidism of GD.

## 5 Treatment

Upon admission, she was treated with 2500 mL 0.9% NaCl and 20 mg furosemide twice daily by intravenous infusion. Methimazole (10 mg three times) and propranolol (10 mg three times) were added to control her hyperthyroidism. Also, febuxostat was taken to correct hyperuricaemia. Five days later, the serum calcium level did not decline but increased a little; 10u elcatonin was subcutaneously injected, and zoledronic acid injection (100 mL: 5 mg) was also used. The next day, the serum calcium level reduced to 2.7 mmol/L. Her symptoms of anorexia, nausea, and vomiting were substantially alleviated. The thyroid function of FT3 (4.30 pg/mL) and PTH (15.30 pg/mL) got back to normal; FT4 (25.3 ng/L) also decreased. Methimazole was reduced to 10 mg daily, and propranolol was decreased to 5 mg three times daily. Subsequently, she was discharged from our hospital with a serum calcium level of 1.92 mmol/L (Figure 2).

**FIGURE 2:**
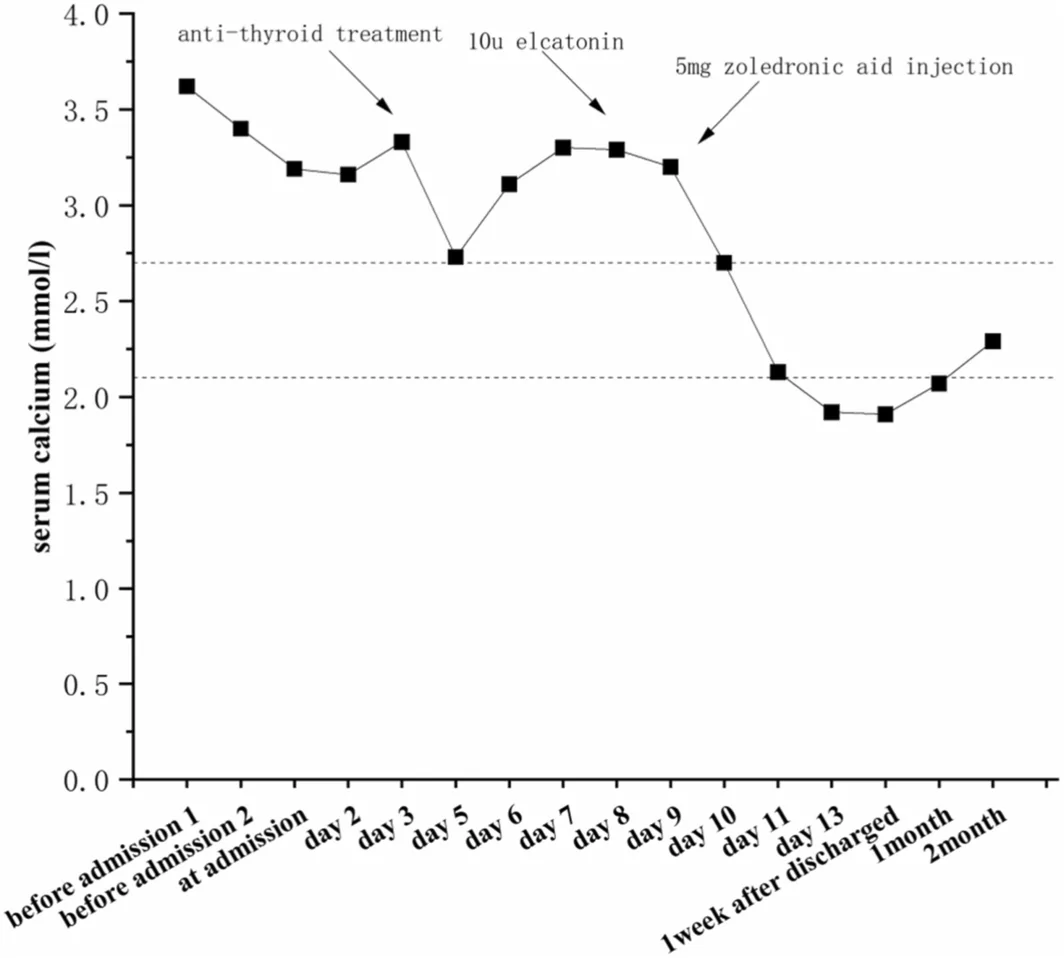
Serum calcium of the patient. Area confined by 2 short dash lines represented the normal range of serum calcium (between 2.1 and 2.6 mmol/L). Black arrows represented the treatment of anti‐thyroid, elcatonin, and zoledronic acid.

## 6 Follow‐Up

One week later, she came for follow‐up. Her serum calcium was 1.91 mmol/L. After 3 weeks, her serum calcium was 2.07 mmol/L, serum FT3 2.59 pg/mL, FT4 6.49 ng/L, TSH < 0.005 uIU/ml, TRAb 34.6 IU/L. She was asked to take methimazole 5 mg daily. 2 months later, her serum calcium was 2.29 mmol/L, serum FT3 4.59 pg/mL, FT4 8.23 ng/L, TSH 0.0277 uIU/ml, TRAb 29.9 IU/L, PTH 51.27 pg/mL, methimazole was decreased to 5 mg qod.

## 7 Discussion

We described a rare case of hypercalcemic crisis induced by hyperthyroidism of GD. The most common etiology of hypercalcemia is primary hyperparathyroidism [8]. In our case, the serum PTH was decreased. Consequently, the hypercalcemia in this patient was PTH‐independent. Furthermore, multiple myeloma was excluded by normal immunoglobulins and SPE. Malignancy related hypercalcemia was excluded by a negative CT (neck, chest, abdomen) scan and normal tumor markers. Vitamin D intoxication was also excluded by normal 25‐OH‐D. Therefore, the etiology of hypercalcemia was considered as GD. However, the serum calcium wasn't declined obviously after anti‐thyroid treatment for 5 days. Due to the plasma half‐life of T4 being 7 days and the release of stored T3, T4 in the thyroid gland taking 2 weeks, we speculated that anti‐thyroid should take longer to decrease the plasma T3, T4 level. Montefusco et al. [9] treated a patient with new‐onset hyperthyroidism complicated with hypercalcemia. One week later, the serum calcium decreased from 3.27 mmol/L and stabilized at a level below 3.0 mmol/L, and it did not decline and stabilize to the normal range until 2 weeks later. Owing to the patient's complaint of “the anorexia, nausea and vomiting were not alleviated obviously so far”, we made a decision to give 10u elcatonin and 5 mg zoledronic acid injection in the hope of decreasing the serum calcium rapidly. The patient's serum calcium decreased to 2.7 mmol/L the next day, and her uncomfortable symptoms were relieved accordingly.

The underlying mechanism of hyperthyroidism‐induced hypercalcemia is not exhaustively understood. Researchers found the serum concentrations of interleukin‐6 (IL‐6) in GD patients were are significantly higher than those in controls. Hyperthyroidism could increase IL‐6 to enhance osteoclastic activity [10, 11]. But the clinical laboratory of our hospital cannot detect the IL‐6. Others proposed increased bone turnover contribute to the increased adrenergic tone in hyperthyroidism [12]. Hyperthyroidism induces high bone turnover with a shortened remodeling time, which causes an increased release of calcium into the blood circulation [13]. Studies indicated increased serum alkaline phosphatase (ALP) levels, an activity marker of osteoblast, were found in approximately 50% of patients with hyperthyroidism complicated by hypercalcaemia [14, 15]. In our case the ALP level was increased mildly. Previous researches showed that serum sclerostin, a wingless/integrated (Wnt) inhibitor, was higher in hyperthyroid versus euthyroid state. Inhibition of the Wnt pathway could cause bone loss [16, 17, 18].

Hyperthyroidism of GD is a rare but important cause of severe hypercalcemia, or hypercalcemic crisis. In patients with hypercalcemia of unknown etiology, hyperthyroidism of GD should be considered. Promptly treatment of hypercalcemia is critical to relieve the symptoms and save patient's life. Anti‐thyroid treatment takes effective control of the hyperthyroidism, which also can strengthen the maintenance of serum calcium level.

## Author Contributions

**Suxin Wang:** writing – original draft, writing – review and editing. **Jing Wang:** supervision.

## Funding

The authors have nothing to report.

## Disclosure

Patient perspective: I often felt anorexic, nauseous, and sick, and lost weight of 10 kg before admission. After the treatment, I felt alleviated and could eat food without vomiting.

## Ethics Statement

The authors have nothing to report.

## Consent

Written informed consent was obtained from the patient for publication of this case report and any accompanying images. A copy of the written consent is available for review by the Editor‐in‐Chief of this journal.

## Conflicts of Interest

The authors declare no conflicts of interest.

## Data Availability

The data that support the findings of this study are available from the corresponding author upon reasonable request.
